# Partial mitochondrial DNA sequences suggest the existence of a cryptic species within the Leucosphyrus group of the genus *Anopheles *(Diptera: Culicidae), forest malaria vectors, in northern Vietnam

**DOI:** 10.1186/1756-3305-3-41

**Published:** 2010-04-30

**Authors:** Kohei Takenaka Takano, Ngoc Thi Hong Nguyen, Binh Thi Huong Nguyen, Toshihiko Sunahara, Michio Yasunami, Manh Duc Nguyen, Masahiro Takagi

**Affiliations:** 1 Institute of Tropical Medicine (NEKKEN), Nagasaki University, Sakamoto, Nagasaki 852-8523, Japan; 2 National Institute of Malariology, Parasitology and Entomology (NIMPE), BC 10-200, Tu Liem, Hanoi, Vietnam; 3 Graduate School of Biomedical Sciences, Nagasaki University

## Abstract

**Background:**

During the last decade, Southeast Asian countries have been very successful in reducing the burden of malaria. However, malaria remains endemic in these countries, especially in remote and forested areas. The Leucosphyrus group of the genus *Anopheles *harbors the most important malaria vectors in forested areas of Southeast Asia. In Vietnam, previous molecular studies have resulted in the identification of only *Anopheles dirus sensu stricto *(previously known as *An. dirus *species A) among the Leucosphyrus group members. However, Vietnamese entomologists have recognized that mosquitoes belonging to the Leucosphyrus group in northern Vietnam exhibit morphological characteristics similar to those of *Anopheles takasagoensis*, which has been reported only from Taiwan. Here, we aimed to confirm the genetic and morphological identities of the members of the Leucosphyrus group in Vietnam.

**Results:**

In the molecular phylogenetic trees reconstructed using partial *COI *and *ND6 *mitochondrial gene sequences, samples collected from southern and central Vietnam clustered together with GenBank sequences of *An. dirus *that were obtained from Thailand. However, samples from northern Vietnam formed a distinct clade separated from both *An. dirus *and *An. takasagoensis *by other valid species.

**Conclusions:**

The results suggest the existence of a cryptic species in northern Vietnam that is morphologically similar to, but phylogenetically distant from both *An. dirus *and *An. takasagoensis*. We have tentatively designated this possible cryptic species as *Anopheles *aff. *takasagoensis *for convenience, until a valid name is assigned. However, it is difficult to distinguish the species solely on the basis of morphological characteristics. Further studies on such as karyotypes and polytene chromosome banding patterns are necessary to confirm whether *An*. aff. *takasagoensis *is a valid species. Moreover, studies on (1) the geographic distribution, which is potentially spreading along the Vietnam, China, Laos, and Myanmar borders; (2) morphological and ecological characteristics; and (3) vectorial capacity of this newly identified cryptic species of *An. dirus*, which is one of the most important malaria vectors in the mainland of Southeast Asia, are necessary for planning efficient malaria vector control programs in this region.

## Background

During the last decade, mainland Southeast Asian countries (i.e., Cambodia, Laos, Myanmar, Thailand, and Vietnam) have been very successful in reducing the burden of malaria [1]. Their main strategies included prompt diagnosis and treatment and widespread coverage of vector control through insecticide-treated nets and indoor residual spraying [2]. Malaria, however, has not yet completely disappeared and remains endemic in these countries. In remote and forested areas, the transmission rates are still high because of complex interactions between vectors, humans, and environmental factors [3], [4]. Indoor residual spraying is ineffective against vectors that rest outdoors after feeding and vectors encountered outdoors [5], and bed nets are not easily adaptable to the lifestyle of forest workers [4]. Environmental modifications that affect the distribution and abundance of vectors lead to changes in malaria transmission [6]. Under these conditions, accurate species identification is essential in vector control.

The Leucosphyrus group consists of 20 formally described species and 2 informal forms, and its members are distributed in the Oriental region [7-9]. The Dirus complex of the Leucosphyrus group includes the most important malaria vectors in forested areas in mainland Southeast Asia [9], [10]. *Anopheles dirus *Peyton and Harrison, 1979 was first separated from *Anopheles balabacensis *Baisas, 1936 [11]. Shortly thereafter, *Anopheles takasagoensis *Morishita, 1946 was elevated to species status from a synonym of *An. balabacensis *on the basis of cross-mating, cytogenetic, and morphological evidence [12]. These findings implied that *An. balabacensis*, which until that time was considered to be the primary vector of human malaria in an area stretching from east India to the Philippines, is not a single species but a complex of three or more species [9]. Mainly on the basis of cross-mating and cytogenetic experiments, subsequent intensive studies [8], [13-18] revealed that *An. dirus *also exists as a species complex that includes at least seven species: *An. dirus sensu stricto *(previously known as *An. dirus *species A); *Anopheles cracens *Sallum and Peyton, 2005 (species B); *Anopheles scanloni *Sallum and Peyton, 2005 (species C); *Anopheles baimaii *Sallum and Peyton, 2005 (species D); *Anopheles elegans *(James), 1903 (species E); *Anopheles nemophilous *Peyton and Ramalingam, 1988 (species F); and *An. takasagoensis*. At least two species in the Dirus complex, namely, *An. dirus *and *An. baimaii*, are recognized as major malaria vectors [9], [10]. *Anopheles balabacensis *is now classified into the Leucosphyrus complex [19] with *Anopheles leucosphyrus *Dönitz, 1901, *Anopheles latens *Sallum and Peyton, 2005, and *Anopheles introlatus *Colless, 1957 [7-9] (see also Table 1).

**Table 1:** In-group and out-group data available from the International Nucleotide Sequence Database with internal classification of the Leucosphyrus group.

	Accession numbers				
				
**Specific name_ID in Sallum *et al*. **[22]	*COI*	*ND6*	Haplo-type	Complex	Subgroup
*latens*_2	DQ897936	DQ899796	23	Leucosphyrus	
*latens*_4	DQ897937	DQ899797	24		
*leucosphyrus*_1	DQ897938	DQ899798	25		
*leucosphyrus*_2	DQ897939	DQ899799	25		
*balabacensis*_1	DQ897940	DQ899800	26		
*balabacensis*_2	DQ897941	DQ899801	27		
*balabacensis*_3	DQ897942	DQ899802	28		
	
*dirus*_3	DQ897943	DQ899803	29	Dirus	Leucosphyrus
*dirus*_4	DQ897944	DQ899804	7		
*dirus*_5	DQ897945	DQ899805	7		
*dirus*_6	DQ897946	DQ899806	7		
*cracens*_1	DQ897947	DQ899807	30		
*cracens*_2	DQ897948	DQ899808	31		
*scanloni*_2	DQ897949	DQ899809	32		
*scanloni*_4	DQ897950	DQ899810	33		
*scanloni*_5	DQ897951	DQ899811	34		
*baimaii*_2	DQ897952	DQ899812	6		
*baimaii*_3	DQ897953	DQ899813	7		
*baimaii*_4	DQ897954	DQ899814	7		
*baimaii*_5	DQ897955	DQ899815	35		
*baimaii*_6	DQ897956	DQ899816	36		
*elegans*_1	DQ897957	DQ899817	37		
*elegans*_3	DQ897958	DQ899818	37		
*nemophilous*_1	DQ897959	DQ899819	38		
*nemophilous*_3B	DQ897960	DQ899820	39		
*nemophilous*_4	DQ897961	DQ899821	40		
*takasagoensis*_1	DQ897962	DQ899822	41		
*takasagoensis*_2	DQ897963	DQ899823	41		
*takasagoensis*_3	DQ897964	DQ899824	42		

*mirans*_1	DQ897965	DQ899825	43		Hackeri
*mirans*_3	DQ897966	DQ899826	44		
*sulawesi*	DQ897967	DQ899827	45		

*macarthuri*_1	DQ897968	DQ899828	46		Riparis
*macarthuri*_2	DQ897969	DQ899829	47		
*macarthuri*_3	DQ897970	DQ899830	48		
*macarthuri*_5	DQ897971	DQ899831	48		
*macarthuri*_6	DQ897972	DQ899832	48		

*gambiae*	L20934	L20934	49	(Outgroup)	
*quadrimaculatus *A	NC_000875	NC_000875	50		
*albimanus*	AF417695	U35259	51		
*aquasalis*	AF417697	U35260	52		

*Anopheles gambiae*, *An. quadrimaculatus *A, *An. albimanus*, and *An. aquasalis *were assigned into an out-group, and the remaining species, as well as samples obtained in the present study, were assigned into an in-group.

Among the members of the Leucosphyrus group, only *An. dirus sensu strict *has been found in Vietnam in previous molecular studies (reviewed in [20]). However, a member of the Leucosphyrus group in northern Vietnam had been identified as *An. takasagoensis *by some Vietnamese entomologists for the last 30 years [21-23]. Here, we conducted a molecular study on members of the Leucosphyrus group in Vietnam to confirm their genetic identity.

Manguin *et al*. [24] analyzed several specimens collected in 1970 from Ninh Bình Province, northern Vietnam, which exhibited adult and larval characteristics of both *An. dirus *and *An. takasagoensis*. They succeeded in sequencing mitochondrial Cytochrome *c *oxidase subunit I (*COI*) and ribosomal DNA internal transcribed spacer 2 (*ITS2*) of one of the specimens and identified it as *An. dirus*, whereas they have not yet deposited the sequence into the DDBJ/EMBL/GenBank database. They noted, "This casts doubt on the reported occurrence of *An. takasagoensis*...in northern Vietnam, but additional material needs to be collected and analysed before it will be known for certain whether the distribution of this species is limited to Taiwan." Here, we conducted a molecular study on members of the Leucosphyrus group in Vietnam to confirm their genetic identity.

Sallum *et al*. [25] conducted a molecular phylogenetic study of the Leucosphyrus group. For comparison, we chose the same molecular markers: the partial sequences of *COI *(221 base pair (bp)) and NADH dehydrogenase subunit 6 (*ND6*: 349 bp) among mitochondrial genes. The advantage of using the same molecular markers is that Sallum *et al*. [25] assessed 13 of 20 species of the Leucosphyrus group, including all the members of the Dirus complex. The disadvantages are that these markers cannot distinguish between *An. dirus *and *An. baimaii *and that the phylogenetic relationship within the group remains ambiguous, presumably because of the short length of the sequences analyzed (570 bp in total). However, to date, this is the only available molecular information covering the group but is still informative for distinguishing species (except *An. dirus *and *An. baimaii*).

## Methods

### Mosquito collection and preliminary identification

**Figure 1:**
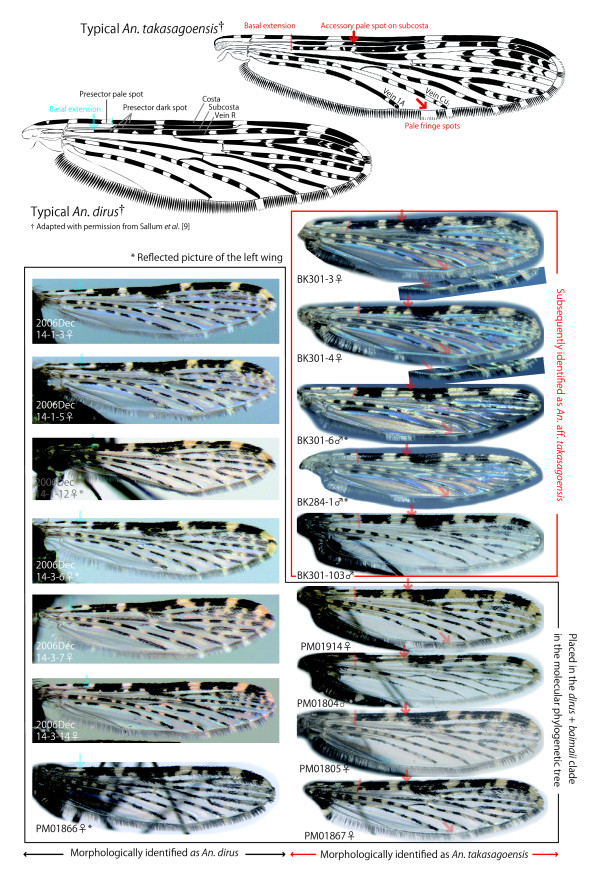
**Comparison of wing-spot patterns (dorsal view)**. Typical *An. takasagoensis *(upper right), typical *An. diru*s (upper left, both adapted with permission from Sallum *et al*. [9]), *An. dirus *analyzed in this study (black border), and *An*. aff. *takasagoensis *(red border). *Anopheles *aff. *takasagoensis *exhibited the same spot patterns as typical *An. takasagoensis*: presector dark spot on vein R that does not extend or barely extends basally beyond the presector pale spot on the costa, a pale fringe spot between veins 1A and Cu_2_, and an accessory sector pale spot on the subcosta. Some samples from Nghệ An Province (PM01914, PM01804, PM01805, and PM01867) also exhibited spot patterns similar to those of *An. takasagoensis*, but they were identified as *An. dirus *by molecular analyses.

In 2008, we conducted field sampling in Bắc Kạn Province for one week and collected 11 larvae (but no adults) of the Leucosphyrus group, eight of which were analyzed in this study (Tables 2 and 3). These larvae were collected from partially or heavily shaded small pools near the starting points (seepage) of small streams in hilly areas covered with secondly evergreen forests. The water of the larval habitats was clear and not running. Other larval and female-adult samples were collected from various parts of Vietnam (Table 1). Collected larvae were reared to obtain adult specimens. Some samples were provided by collaborative entomologists, and a Hai Nan Island (China) strain of *An. dirus *maintained at the National Institute of Malariology, Parasitology and Entomology was also analyzed. The adult samples were tentatively identified as *An. takasagoensis *if they had more than one of the following three morphological characteristics (we followed the terminology reported in [26], [27] as well as in [9]): a presector dark spot on vein R not or barely extending basally beyond the presector pale spot on the costa (basal extension typically occurs in *An. dirus*), a pale fringe spot between veins 1A and Cu_2 _present on at least one wing (absent in *An. dirus*), and an accessory sector pale spot on the subcosta (absent in *An. dirus*) (Table 1 and Figure 1).

### DNA extraction

The specimens had been stored dry at room temperature or below -20°C for up to 7 years prior to DNA extraction. Depending on the condition of each specimen, we used a single leg or a combination of a single leg and some other body parts (i.e., additional legs, a wing, or a head, but not female abdomens, the spermathecae of which might include sperm from mating). We extracted DNA using the REDExtract-N-Amp™ Tissue PCR Kit (Sigma-Aldrich) with a modification of the manufacturer's protocol for animal tissues. Extraction Solution and Tissue Preparation Solution were mixed in a 4:1 ratio. We added 20 μl of the mixture per leg and homogenized them in a microtube using the tip of a pipette. After 10 min of incubation at room temperature, the samples were incubated at 95°C for 3 min. We added 16 μl of Neutralization Solution B per 20 μl of the homogenized sample and mixed them by vortexing. The neutralized tissue extract was centrifuged at 10,000 *g *for 1 min, and the supernatant was added to a new microtube and stored at 4°C until used for PCR reactions.

### Markers and primers used in this study

We selected molecular markers and primers according to Sallum *et al *[22]. Amplification with the primers UEA9.2 (5'-cta aca ttt ttt cct caa cat ttt tta gg-3') and UEA10.2 (5'-tta tta gtt aat aay ggt art tct g-3') yielded a 221-bp product (excluding the primers), partial sequence of the *COI *gene. Amplification of the primers ND6.F2 (5'-ttg gwc gta awg gwc cat aaa a-3') and ND6.R3 (5'-car gaa tyt atg taa aaa cat ttt g-3') resulted in a product of 349 bp (excluding the primers), part of the *ND6 *gene.

### PCR, sequencing, and alignment

We modified the PCR protocol of Sallum *et al*. [25]. For *COI*, each 20-μl PCR reaction contained 2 μl of 10×EX Taq buffer (TaKaRa, Japan), 0.2 μM of dNTP, 0.5 μM of UEA9.2 primer, 1.0 μM of UEA10.2 primer, 0.1 μl of EX Taq^® ^Hot Start Version (TaKaRa), and 1.0 to 2.0 μl of DNA extract. The reaction mixture for *ND6 *was the same as that for *COI*, except that 1.0 μM each of ND6F.2 and ND6.R3 primer was added instead of UEA9.2 and UEA10.2. ASTEC PC320 and PC816 thermal cyclers were used. The thermal cycling profile for *COI *consisted of 5 cycles of 30 s at 94°C, 30 s at 37°C, and 30 s at 72°C, followed by 40 cycles of 30 s at 94°C, 30 s at 47°C, and 30 s at 72°C, with a final extension of 2 min at 72°C. The profile for *ND6 *consisted of 5 cycles of 30 s at 94°C, 30 s at 37°C, and 30 s at 72°C, followed by 45 cycles of 30 s at 94°C, 30 s at 49°C, and 30 s at 72°C, with a final extension of 2 min at 72°C. The PCR product was separated on a 2% agarose gel and visualized by ethidium bromide staining. Fragment sizes and product density were estimated by comparison with molecular weight standards. We purified the PCR products using ExoSAP-IT (GE Healthcare Japan). We diluted ExoSAP-IT 10 times with Milli-Q water, added 2 μl of the dilution to 5 μl of the PCR product, incubated the solution at 37°C for 30 min, and then inactivated enzymes by incubating at 80°C for 15 min. Sequence reactions were carried out on both strands of DNA using the primers listed above and the ABI BigDye^® ^terminator Cycle Sequencing Kit v3.1 (Applied Biosystems). The reaction products were purified by ethanol precipitation and resolved in Hi-Di™ Formamide following the manufacturer's protocol, and the sequences were determined with an ABI PRISM 3730 Genetic Analyzer. Complimentary strands were combined into consensus sequences, and questionable base calls were corrected manually by comparison with the original waveform. When the correction of the questionable base call was difficult, the site was recorded as missing data. We concatenated the *COI *and *ND6 *sequences and identified 22 unique sequences, i.e., haplotypes (Tables 2 and 3).

**Table 2:** Specimens used in this study (to be continued).

Specimen ID	Morphological identification	Sex	Collection date	Collector	Specimen ID	Latitude	Longitude	Place name in Vietnam	
								
								Area	Province
S1	*An. dirus*	M	24-x-2005	Sunahara T. *et al*.	S1	11°59'36.12"N	107°18'13.14"E	Southern Vietnam	Bình Phuớc
S2	*An. dirus*	M	24-x-2005	Sunahara T. *et al*.	S2	11°59'36.12"N	107°18'13.14"E		Bình Phuớc
S5	*An. dirus*	F	25-x-2005	Sunahara T. *et al*.	S5	11°59'30.84"N	107°18'12.12"E		Bình Phuớc
S6	*An. dirus*	F	25-x-2005	Sunahara T. *et al*.	S6	11°59'30.84"N	107°18'12.12"E		Bình Phuớc
S7	*An. dirus*	F	8-xi-2005	Sunahara T. *et al*.	S7	11°05'26.35"N	107°53'59.43"E		Bình Thuận
S8	*An. dirus*	F	8-xi-2005	Sunahara T. *et al*.	S8	11°05'26.35"N	107°53'59.43"E		Bình Thuận
S9	*An. dirus*	M	8-xi-2005	Sunahara T. *et al*.	S9	11°05'26.35"N	107°53'59.43"E		Bình Thuận
S11	*An. dirus*	F	13-xii-2006	Sunahara T. *et al*.	S11	11°42'49.58"N	106°56'02.51"E		Bình Phuớc
S12	*An. dirus*	F	19-xii-2006	Sunahara T. *et al*.	S12	11°42'47.06"N	106°56'54.08"E		Bình Phuớc
S13	*An. dirus*	F	19-xii-2006	Sunahara T. *et al*.	S13	11°42'47.06"N	106°56'54.08"E		Bình Phuớc
V24	*An. dirus*	F	2002	Nguyen D. M. *et al*.	V24	12°22'21.00"N	109°05'4.12"E		Khánh Hòa
V25	*An. dirus*	F	2002	Nguyen D. M. *et al*.	V25	12°22'21.00"N	109°05'4.12"E		Khánh Hòa
V27	*An. dirus*	F	2002	Nguyen D. M. *et al*.	V27	12°22'21.00"N	109°05'4.12"E		Khánh Hòa
V43	*An. dirus*	F	2003	Nguyen D. M. *et al*.	V43				Bình Phuớc
V51	*An. dirus*	F	2007	Vu Dinh Chu *et al*.	V51	13°08'N	108°50'E		Phú Yên
V52	*An. dirus*	F	2007	Vu Dinh Chu *et al*.	V52	13°08'N	108°50'E		Phú Yên
V53	*An. dirus*	F	2007	Vu Dinh Chu *et al*.	V53	13°08'N	108°50'E		Phú Yên
V54	*An. dirus*	F	2007	Vu Dinh Chu *et al*.	V54	13°08'N	108°50'E		Phú Yên
V71	*An. dirus*	F	2003	Nguyen D. M. *et al*.	V71	11°05'N	107°54'E		Bình Thuận
V72	*An. dirus*	F	2003	Nguyen D. M. *et al*.	V72	11°05'N	107°54'E		Bình Thuận
V73	*An. dirus*	F	2003	Nguyen D. M. *et al*.	V73	11°05'N	107°54'E		Bình Thuận
V74	*An. dirus*	F	2005	Nguyen Van Chau *et al*.	V74	11°30'N	107°20'E		Đống Nai
V76	*An. dirus*	F	2005	Nguyen Van Chau *et al*.	V76	11°30'N	107°20'E		Đống Nai
2006Dec14-1-3	*An. dirus*	F	14-xii-2006	Takano T. K. *et al*.	2006Dec14-1-3	11°42'45.9"N	106°56'01.7"E		Bình Phuớc
2006Dec14-1-5	*An. dirus*	F	14-xii-2006	Takano T. K. *et al*.	2006Dec14-1-5	11°42'45.9"N	106°56'01.7"E		Bình Phuớc
2006Dec14-1-12	*An. dirus*	F	14-xii-2006	Takano T. K. *et al*.	2006Dec14-1-12	11°42'45.9"N	106°56'01.7"E		Bình Phuớc
2006Dec14-3-6	*An. dirus*	F	14-xii-2006	Takano T. K. *et al*.	2006Dec14-3-6	11°42'57.9"N	106°56'01.2"E		Bình Phuớc
2006Dec14-3-7	*An. dirus*	F	14-xii-2006	Takano T. K. *et al*.	2006Dec14-3-7	11°42'57.9"N	106°56'01.2"E		Bình Phuớc
2006Dec14-3-14	*An. dirus*	F	14-xii-2006	Takano T. K. *et al*.	2006Dec14-3-14	11°42'57.9"N	106°56'01.2"E		Bình Phuớc
							
Hai Nan strain	*An. dirus*		2008		Hai Nan strain	19° 7'25.03"N	109°34'4.05"E	China	
							
PM01866	*An. dirus*	F	x-2006	Vu Duc Chinh *et al*.	PM01866	19°02'N	104°48'E	Central Vietnam	Nghệ An
									
PM01867	*An. takasagoensis*	F	x-2006	Vu Duc Chinh *et al*.	PM01867	19°02'N	104°48'E		Nghệ An
PM01804	*An. takasagoensis*	M	x-2006	Vu Duc Chinh *et al*.	PM01804	19°02'N	104°48'E		Nghệ An
PM01805	*An. takasagoensis*	F	x-2006	Vu Duc Chinh *et al*.	PM01805	19°02'N	104°48'E		Nghệ An
PM01914	*An. takasagoensis*	F	x-2006	Vu Duc Chinh *et al*.	PM01914	19°02'N	104°48'E		Nghệ An
							
BK101	*An. takasagoensis*	F	ix-2007	Le Xuan Hoi *et al*.	BK101	22°05'59.21"N	106°01'26.00"E	Northern Vietnam	Bắc Kạn
BK284-2	*An. takasagoensis*	M	8-x-2008	Nguyen D. M.	BK284-2	22°05'54.29"N	106°01'20.71"E		Bắc Kạn
BK301-6	*An. takasagoensis*	M	10-x-2008	Hoang Van Tan *et al*.	BK301-6	22°05'40.31"N	106°02'17.52"E		Bắc Kạn
BK301-7	*An. takasagoensis*	M	10-x-2008	Hoang Van Tan *et al*.	BK301-7	22°05'40.31"N	106°02'17.52"E		Bắc Kạn
BK301-103	*An. takasagoensis*	M	14-x-2008	Tsuzuki ataru *et al*.	BK301-103	22°05'40.31"N	106°02'17.52"E		Bắc Kạn
BK284-1	*An. takasagoensis*	M	8-x-2008	Nguyen D. M.	BK284-1	22°05'54.29"N	106°01'20.71"E		Bắc Kạn
BK301-3	*An. takasagoensis*	F	10-x-2008	Hoang Van Tan *et al*.	BK301-3	22°05'40.31"N	106°02'17.52"E		Bắc Kạn
BK301-4	*An. takasagoensis*	F	10-x-2008	Hoang Van Tan *et al*.	BK301-4	22°05'40.31"N	106°02'17.52"E		Bắc Kạn

**Table 3:** Specimens used in this study (continued).

	Place name in Vietnam					**DDBJ Accession No**.		
					
Specimen ID	District	Commune	Others	Remarks	Haplo-type	*COI*	*ND6*	Molecular identification
S1	Bù Đăng	Đắk Nhau	Đắk Liên	Larval collection	1	AB518499	AB518539	*An. dirus*
S2	Bù Đăng	Đắk Nhau	Đắk Liên	Larval collection	2	AB518500	AB518540	*An. dirus*
S5	Bù Đăng	Đắk Nhau	Đắk Liên	Larval collection	3	AB518501	AB518541	*An. dirus*
S6	Bù Đăng	Đắk Nhau	Đắk Liên	Larval collection	3	AB518502	AB518542	*An. dirus*
S7	Hàm Thuận Nam	Mỹ Thanh		Larval collection	4	AB518503	AB518543	*An. dirus*
S8	Hàm Thuận Nam	Mỹ Thanh		Larval collection	5	AB518504	AB518544	*An. dirus*
S9	Hàm Thuận Nam	Mỹ Thanh		Larval collection	5	AB518505	AB518545	*An. dirus*
S11	Phuớc Long	Phú Riêng	Phú Thuận	Indoor light trap	6	AB518506	AB518546	*An. dirus*
S12	Phuớc Long	Phú Riêng	Phú Thuận	Indoor light trap	7	AB518507	AB518547	*An. dirus*
S13	Phuớc Long	Phú Riêng	Phú Thuận	Indoor light trap	6	AB518508	AB518548	*An. dirus*
V24	Khánh Vinh	Khánh Phú	a forest near Ngã Hai village	Human landing catch	7	AB518509	AB518549	*An. dirus*
V25	Khánh Vinh	Khánh Phú	a forest near Ngã Hai village	Human landing catch	7	AB518510	AB518550	*An. dirus*
V27	Khánh Vinh	Khánh Phú	a forest near Ngã Hai village	F1 from an adult female	7	AB518511	AB518551	*An. dirus*
V43					8	AB518512	AB518552	*An. dirus*
V51	Son Hòa	Ea Chà Rang	Kiến Thiết village	Human landing catch	9	AB518513	AB518553	*An. dirus*
V52	Son Hòa	Ea Chà Rang	Kiến Thiết village	Human landing catch	9	AB518514	AB518554	*An. dirus*
V53	Son Hòa	Ea Chà Rang	Kiến Thiết village	Human landing catch	9	AB518515	AB518555	*An. dirus*
V54	Son Hòa	Ea Chà Rang	Kiến Thiết village	Human landing catch	9	AB518516	AB518556	*An. dirus*
V71	Hàm Thuận Nam	Mỹ Thanh		Human landing catch	10	AB518517	AB518557	*An. dirus*
V72	Hàm Thuận Nam	Mỹ Thanh		Human landing catch	10	AB518518	AB518558	*An. dirus*
V73	Hàm Thuận Nam	Mỹ Thanh		Human landing catch	7	AB518519	AB518559	*An. dirus*
V74	Tận Phú	Đắc Lua	Cát Tiên National Park	Human landing catch	5	AB518520	AB518560	*An. dirus*
V76	Tận Phú	Đắc Lua	Cát Tiên National Park	Human landing catch	11	AB518521	AB518561	*An. dirus*
2006Dec14-1-3	Phuớc Long	Phú Riêng	Phú Thuận	Larval collection	12	AB518522	AB518562	*An. dirus*
2006Dec14-1-5	Phuớc Long	Phú Riêng	Phú Thuận	Larval collection	10	AB518523	AB518563	*An. dirus*
2006Dec14-1-12	Phuớc Long	Phú Riêng	Phú Thuận	Larval collection	7	AB518524	AB518564	*An. dirus*
2006Dec14-3-6	Phuớc Long	Phú Riêng	Phú Thuận	Larval collection	7	AB518525	AB518565	*An. dirus*
2006Dec14-3-7	Phuớc Long	Phú Riêng	Phú Thuận	Larval collection	13	AB518526	AB518566	*An. dirus*
2006Dec14-3-14	Phuớc Long	Phú Riêng	Phú Thuận	Larval collection	7	AB518527	AB518567	*An. dirus*
Hai Nan strain		Reared strain in NIMPE, originated from Hai Nan Island, China			9	AB518528	AB518568	*An. dirus*
PM01866	Con Cuông	Chi Khê	Pù Mát National Forest	Larval collection	14	AB518529	AB518569	*An. dirus*
PM01867	Con Cuông	Chi Khê	Pù Mát National Forest	Larval collection	15	AB518530	AB518570	*An. dirus*
PM01804	Con Cuông	Chi Khê	Pù Mát National Forest	Larval collection	16	AB518531	AB518571	*An. dirus*
PM01805	Con Cuông	Chi Khê	Pù Mát National Forest	Larval collection	16	AB518532	AB518572	*An. dirus*
PM01914	Con Cuông	Chi Khê	Pù Mát National Forest	Larval collection	17	AB518533	AB518573	*An. dirus*
								
BK101	Na Rì	Quang Phong	Na Ca village	Collected at buffalo hat	18	AB518534	AB518574	*An. *aff. *takasagoensis*
BK284-2	Na Rì	Quang Phong	Na Ca village	Larval collection	19	AB518535	AB518575	*An. *aff. *takasagoensis*
BK301-6	Na Rì	Quang Phong	Na Ca village	Larval collection	20	AB518536	AB518576	*An. *aff. *takasagoensis*
BK301-7	Na Rì	Quang Phong	Na Ca village	Larval collection	21	AB518537	AB518577	*An. *aff. *takasagoensis*
BK301-103	Na Rì	Quang Phong	Na Ca village	Larval collection	22	AB518538	AB518578	*An. *aff. *takasagoensis*
BK284-1	Na Rì	Quang Phong	Na Ca village	Larval collection	Not analyzed but wing spots are shown in Figure 2.			
BK301-3	Na Rì	Quang Phong	Na Ca village	Larval collection	Not analyzed but wing spots are shown in Figure 2.			
BK301-4	Na Rì	Quang Phong	Na Ca village	Larval collection	Not analyzed but wing spots are shown in Figure 2.			

Further, we obtained the GenBank sequences of members of the Leucosphyrus group (in-group, 37 samples) and those of four other *Anopheles *species (out-group, Table 1). Sallum *et al*. [25] deposited the *COI *sequences that include the sequence of the UEA9.2 primer (29 bp) in GenBank and reconstructed phylogenetic trees on the basis of these sequences. We excluded the UEA9.2 primer sequence from our analysis. There were no insertions or deletions in these sequences; however, the sequences BK101, BK284-2, BK301-103 (Table 2), *balabacensis*_3, *dirus*_3, and *baimaii*_6 (Table 1) had 8-87 missing sites at the 3'- or 5'-end of either the *COI *or *ND6 *sequences (Additional files 1, 2 and 3). Finally, we obtained 52 unique sequences and assigned a haplotype to each of them (Tables 1, 2 and 3 and Additional files 1, 2 and 3).

### Molecular phylogeny

The neighbor-joining (NJ) and maximum parsimony (MP) methods were performed with the MEGA4 software [28]. All codon positions were included, and all ambiguous sites were treated as missing data. The resultant trees were rooted using the out-group. In the NJ phylogenetic reconstruction, the evolutionary distances were computed using the Jukes-Cantor method. All sites containing missing data were eliminated only in pairwise sequence comparisons (pairwise deletion option). To assess the reliability of the NJ tree, the bootstrap test and the interior branch test were performed with 2,000 replicates. In the MP phylogenetic reconstruction, the most parsimonious trees were obtained using the close-neighbor-interchange algorithm at search level 3, in which the initial trees were obtained by random addition of sequences (10,000 replicates). There were 570 sites in the final dataset, of which 137 were parsimony informative. The consensus tree was generated from the 3517 most parsimonious trees. Branches corresponding to partitions reproduced in less than 50% of trees were collapsed. Branch lengths were calculated using the average pathway method and are expressed in units of the number of changes over the whole sequence. The percentages of parsimonious trees in which the associated taxa clustered together are shown next to the branches.

## Results

By the morphological examination, all the samples collected from Bắc Kạn Province in northern Vietnam and four of five samples from Nghệ An Province in central Vietnam were tentatively identified as *An. takasagoensis *(Tables 2 and 3 and Figure 1).

**Figure 2:**
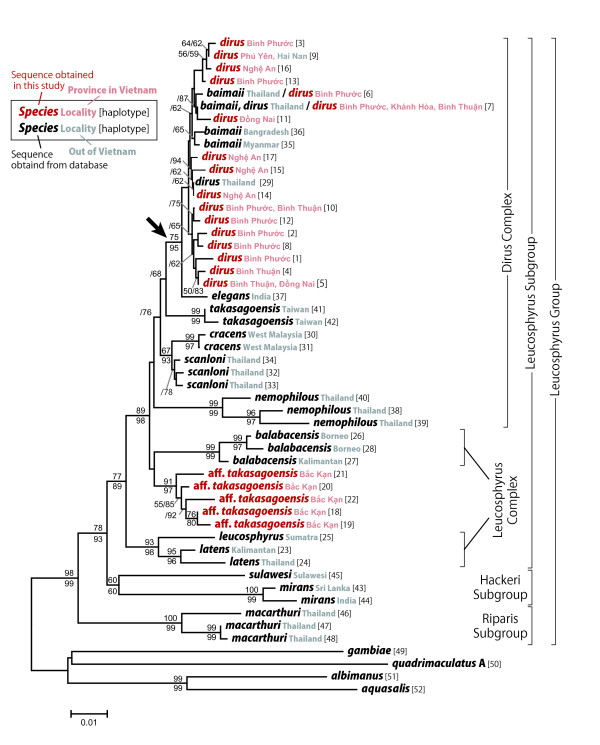
**Neighbor-joining tree with traditional classification**. The evolutionary distances were computed using the Jukes-Cantor method based on concatenated sequences of partial *COI *(221 bp) and *ND6 *(349 bp) mitochondrial genes. All positions containing missing data were eliminated only in pairwise sequence comparisons (Pairwise deletion option). The bootstrap test and the interior branch test were performed with 2,000 replicates, respectively, and each value equal to or above 50% is shown above (bootstrap value) and below (interior branch test support) the branches.

In the molecular phylogenetic reconstruction using the NJ method (Figure 2), haplotypes 18-22 of the Bắc Kạn samples formed a distinct clade with high bootstrap (91%) and interior branch test (97%) support; this clade was separated from haplotypes of both *An. takasagoensis* and *An. dirus*. This clade then clustered with haplotypes 26-28 of *An. balabacensis*, but the bootstrap and interior branch test support were lower, with values less than 50%. Other haplotypes obtained in the present study (haplotypes 1-17), including those of the Nghệ An samples, were clustered together with those of *An. dirus *from Thailand and *An. baimaii *from Thailand, Myanmar, and Bangladesh. The (*An. dirus *+ *An. baimaii*) clade was subsequently clustered with *An. elegans *from India with moderate bootstrap support (75%) and high interior branch test support (95%) (indicated with an arrow in Figure 2). Subsequently, *An. takasagoensis *clustered with the (*An. dirus *+ *An. baimaii *+ *An. elegans*) clade, *An. cracens *clustered with the (*An. dirus *+ *An. baimaii *+ *An. elegans + An. takasagoensis*) clade, and *An. scanloni *clustered with the (*An. dirus *+ *An. baimaii *+ *An. elegans + An. takasagoensis *+ *An. cracens*) clade with moderate to low bootstrap and interior branch test support (Figure 2). The Dirus complex members, *An. balabacensis*, and the Bắc Kạn samples formed a clade with high bootstrap (89%) and interior branch (98%) support. This clade next combined with the (*An. leucosphyrus *+ *An. latens*) clade, and the resultant clade corresponded to the Leucosphyrus subgroup. The Leucosphyrus subgroup clade combined with the Hackeri subgroup clade (*An. sulawesi *+ *An. mirans*), and further combined with the Riparis subgroup clade (*An. macarthuri*). The NJ topology was consistent with the traditional classification of the Leucosphyrus group [9], except that the Leucosphyrus complex were regarded as paraphyletic taxa (Figure 2).

**Figure 3:**
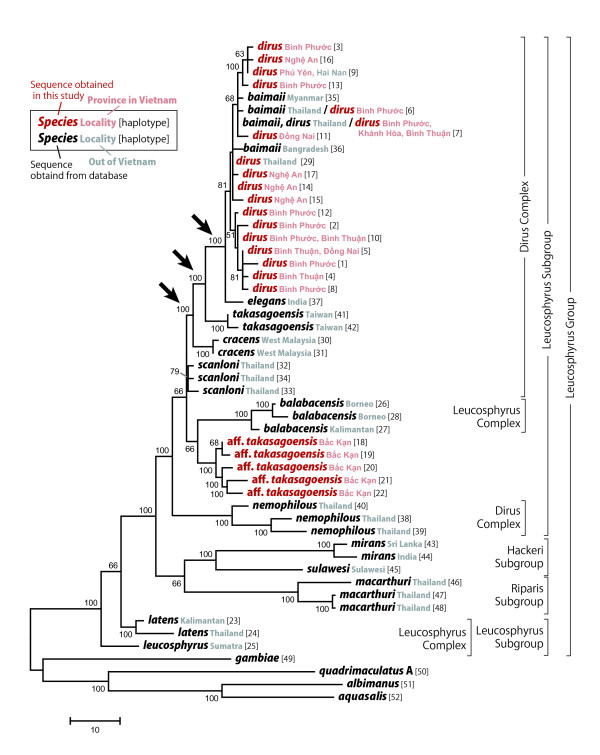
**Maximum parsimony 50%-majority-rule consensus tree with traditional classification**. The consensus tree was generated from the 3517 most parsimonious trees based on the concatenated sequences of partial *COI *(221 bp) and *ND6 *(349 bp) mitochondrial genes. Branches corresponding to partitions reproduced in less than 50% trees are condensed. The percentages of parsimonious trees in which the associated taxa clustered together are shown next to the branches. Branch lengths were calculated using the average pathway method and are expressed in units of the number of changes over the whole sequence (scale bar). *Anopheles gambiae*, *An. quadrimaculatus *A, *An. albimanus*, and *An. aquasalis *are assigned as out-group taxa.

In the MP tree (Figure 3), haplotypes 18-22 from the Bắc Kạn samples also formed a clade with 100% consensus; this clade was separated from the haplotypes of both *An. takasagoensis *and *An. dirus*. The Bắc Kạn haplotypes then clustered with haplotypes 26-28 of *An. balabacensis *with 66% consensus. Other haplotypes obtained in the present study (haplotypes 1-17), including those from the Nghệ An samples, were clustered together with those of *An. dirus *from Thailand and *An. baimaii *from Thailand, Myanmar, and Bangladesh with 81% consensus. Subsequently, the topology ((((*An. dirus*, *An. baimaii*) *An. elegans*) *An. takasagoensis*) *A. cracens*) was supported by 100% consensus (indicated by arrows in Figure 3). This clade clustered with *An. scanloni *and the (*An. balabacensis *+ Bắc Kạn samples) clade with 66% consensus and further combined with the *An. nemophilous *clade with 100% consensus. This clade then combined with the ((*An. sulawesi *+ *An. mirans*: the Hackeri subgroup) + *An. mirans*: the Riparis subgroup) clade with 100% consensus, whereas *An. leucosphyrus *and *An. latens *formed the most basal lineage and second most basal lineage, respectively, in the Leucosphyrus group (Figure 3). Thus, the MP topology was less consistent with the traditional classification of the Leucosphyrus group in that neither the Dirus complex nor the Leucosphyrus complex and the Leucosphyrus subgroup were regarded as monophyletic taxa.

## Discussion

### Recognition of a possible cryptic species

For the last 30 years, Vietnamese medical entomologists [21-23] have noted that mosquitoes belonging to the Leucosphyrus group collected from northern Vietnam exhibited wing-spot patterns that are different from those of *An. dirus *from southern Vietnam, but similar to those of *An. takasagoensis*, which has actually only been found in Taiwan. However, the spot pattern variations partially overlap between *An. dirus *and *An. takasagoensis *so that it is generally difficult to determine these species solely on the basis of morphological characteristics [9]. This seems to be the very reason why foreign scientists recognized that among the Leucosphyrus group members, *An. dirus *is the only species that is distributed in Vietnam.

In the present study, all the haplotypes of the mosquitoes from southern and central Vietnam clustered into the (*An. dirus *+ *An. baimaii*) clade. Although partial sequences of *COI *and *ND6 *in mitochondrial DNA do not provide a clear distinction between *An. dirus *and *An. baimaii *[25], [29], it is reasonable to regard the samples from southern and central Vietnam as *An. dirus *after taking into consideration the well known distributions of *An. dirus *and *An. baimaii *[25], [29] (Molecular identification in Table 1). However, molecular phylogenetic analyses in this study could not resolve population structure of *An. dirus *in Vietnam. This is also the limitation of the molecular markers and beyond the scope of the study so that we refrain from discussing the population structures of *An. dirus *in Vietnam at present.

The haplotypes of samples collected from Bắc Kạn Province in northern Vietnam were clearly separated from those of both *An. dirus *and *An. takasagoensis *in both the NJ and MP trees. In the NJ tree, the Bắc Kạn samples formed a distinct clade with 91% bootstrap and 97% interior branch test support, whereas the (*An. dirus *+ *An. baimaii*) clade formed another clade with *An. elegans *with 75% bootstrap support and 95% interior branch test support (indicated by an arrow in Figure 2). In the MP tree, the Bắc Kạn samples again formed a distinct clade with 100% consensus, whereas the (*An. dirus *+ *An. baimaii*) clade formed another clade with *An. elegans* with 100% consensus, and this clade subsequently formed other clades with *An. takasagoensis *and *An. cracens *with 100% consensuses, respectively (indicated by arrows in Figure 3). These results suggest that the Bắc Kạn samples are distinctly separated from *An. dirus *by at least three valid species--*An. elegans*, *An. takasagoensis*, and *An. cracens*--and from *An. takasagoensis *by at least one valid species--*An. cracens*. The clade consisting of Bắc Kạn samples formed another clade with *An. balabacensis*; however, the reliability of the branch was not high, with 66% consensus in the MP tree and less than 50% bootstrap and interior branch test support in the NJ tree. The overall morphological characteristics of the Bắc Kạn samples, however, were closest to or even indistinguishable from those of *An. takasagoensis *and *An. dirus *but were distinguishable from those of *An. balabacensis *and the other Leucosphyrus group members (Figure 1). Moreover, the distribution of *An. balabacensis *is known to be restricted to the area from the Philippines up to Indonesia.

These results suggest that the mosquito samples obtained from Bắc Kạn Province belong to the Leucosphyrus group but not to *An. dirus*, *An. takasagoensis*, *An. balabacensis*, or any other species in the Leucosphyrus group; thus, these samples seem to represent a newly recognized cryptic species in the Leucosphyrus group. We tentatively designate the possible cryptic species as *Anopheles *aff. *takasagoensis *for convenience, until a valid name is assigned.

### Phylogenetic relationship among the Leucosphyrus group members

In the NJ tree, the possible cryptic species formed a clade together with *An. balabacensis *and members of the Dirus complex with 89% bootstrap and 98% interior branch test support, whereas the other Leucosphyrus complex members, namely, *An. leucosphyrus *and *An. latens*, formed another clade beside the former clade. This topology seems to be consistent with the indications by Sallum *et al*. [25]. They stated that morphological distinction between the Leucosphyrus and the Dirus complexes is problematic because some characters used to define the limits of each species complex are polymorphic. Generally, members of the Leucosphyrus complex can be easily distinguished from those of the Dirus complex by the presence of an accessory sector pale (ASP) wing spot on veins C, subcosta, and R and the absence of pale scales at the base of hind tarsomere 4 [25]. However, *An. balabacensis *is polymorphic for these characters and thus can overlap with members of both the Dirus complex and Leucosphyrus complex [25].

In the MP tree, the (*An*. aff. *takasagoensis *+ *An. balabacensis *+ members of the Dirus complex) clade was also supported by 100% consensus, whereas the topology within the clade was consistent with that observed in the case of the NJ tree only for the (((*An. dirus*, *An. baimaii*) *An. elegans*) *An. takasagoensis*) relationship. Moreover, *An. leucosphyrus *and *An. latens *were separated from the other members of the Leucosphyrus subgroup and formed the most basal lineage and second most basal lineage in the Leucosphyrus group, respectively. This might be partly because of the long-branch attraction, to which the MP method is more sensitive than the NJ method with a corrected distance model is. It is not possible to correct for multiple nucleotide substitutions at the same site in the MP method; this leads to systematic underestimation of the genetic distances. Hence, distant species will either be clustered together or drawn toward the root of the tree [30], [31]. However, this basal positioning of *An. leucosphyrus *and *An. latens *was also reproduced by phylogenetic reconstruction using the maximum likelihood and Bayesian methods in our preliminary analyses (data not shown). This indicates the limitations of the present dataset: the length of the sequence data is limited, and it includes 13 of 20 species in the Leucosphyrus group whereas including all the species from various locality is desirable.

Although the information is limited, we would like to propose following three hypotheses to be tested in the future studies. First, *An. nemophilous *should be removed from the Dirus complex. The remaining members of the Dirus complex are then characterized by morphological characteristics in that pale scales on anterior veins of wing, especially those on presector pale and sector pale spots of the costa, are white and contrasting with other yellowish to golden pale spots on remaining posterior veins [9] (but *An*. aff. *takasagoensis *has the same characteristics). Second, members of the newly hypothesized Dirus complex (*An. dirus*, *An. cracens*, *An. scanloni*, *An. baimaii*, *An. elegans*, and *An. takasagoensis*) and *An. nemophilous*, *An. balabacensis *and *An*. aff. *takasagoensis *further form a distinct taxonomical group that is equivalent to a subgroup. Third, *An. leucosphyrus *and *An. latens *belong to the most basal or even an outer group of the remaining members of the Leucosphyrus subgroup analyzed in this study.

### Morphology of the cryptic species

The cryptic species exhibited morphological characteristics distinguishable from those of typical *An. dirus *in southern Vietnam: the presector dark spot on vein R that does not extend or barely extends basally beyond the presector pale spot on the costa, a pale fringe spot present between veins 1A and Cu_2_, and an accessory sector pale spot on the subcosta on at least one wing.

We must note, however, that these characteristics are still included within the intraspecific morphological variation of *An. dirus*[9]. Samples from Nghệ An Province in central Vietnam exhibited the same morphological characteristics of the cryptic species, but their haplotypes were placed within the monophyletic clade consisting of *An. dirus *haplotypes. The samples collected from Ninh Bình Province in northern Vietnam and analyzed by Manguin *et al*. ([24], mentioned in Background), with morphological characteristics of both *An. dirus *and *An. takasagoensis*, might have been individuals of this *An. dirus *type. The geographical proximity of Ninh Bình and Nghệ An provinces supports this speculation. It is known that wing-spot patterns of *Anopheles *mosquitoes can vary according to temperature and day-length [32]. The wing-spot patterns of *An. dirus *might also vary along with the longitude in Vietnam.

### Distribution of the species

According to the collection records based on identification using wing-spot patterns, populations of the hypothetical cryptic species have been shrinking after the 1970s, presumably because of deforestation in northern Vietnam (NDM, personal observation). Samples of the putative cryptic species have been sporadically collected from central and northern Vietnam. In 1970, 14 larvae were collected from a rice field surrounded by a forest in Cúc Phuong National Park in Ninh Bình Province. The resultant nine larval and five pupal exuviae and nine female-adult specimens are deposited in NIMPE, even though the each exuviae is mounted on a slide grass and the each adult specimens is encapsulated in a glass tube and is not available for genetic analyses. In 1973, less than 10 adult females were collected by human bate from Hòa An District, Cao Báng Province, which is located along the northern border with China (NDM, personal communication). In 2001, the putative cryptic species was collected from Yên Thành Commune, Quang Bình District, Hà Giang Province, which is also located along the northern border with China (Le Xuan Hoi, personal communication). Also in 2001, the putative cryptic species is collected from Chiêng Yên commune, Môc Châu District, Son La Province, which is located along northern-western border with Laos. Other samples are also collected from Trương Son commune, Luong Son District and Phúc San commune, Mai Châu District in Hòa Bình Province in northern Vietnam. Taking the information above and the results of molecular analyses in Manguin *et al*. [24] and the present study into consideration, *An*. aff. *takasagoensis *seems to replace *An. dirus *in the north of Ninh Bình Province (about 20°N). However, it is unclear whether *An. dirus *and *An*. aff. *takasagoensis *are distributed sympatrically. Further confirmation using molecular markers is necessary.

Bắc Kạn Province, from where *An*. aff. *takasagoensis *samples were collected in this study, is located near the border of Vietnam and China. *Anopheles baimaii *occurs in Yunnan Province in China along the borders of Laos and Myanmar [33]. Walton *et al*. [34] showed that the *ITS2 *sequence of the Chinese "species D" (*An. dirus *species D or *An. baimaii*) of Xu and Qu [35] is distinct from that of specimens collected in Thailand and suggested that the Chinese "species D" may represent an unrecognized species of the Dirus complex. We, however, failed to obtain consistent *ITS2 *sequences from our samples over the course of the present study. Confirmation of the genetic identities of *An*. aff. *takasagoensis *and the putative *An. baimaii *from the areas along the Vietnam, China, Laos, and Myanmar borders is also necessary.

### Biology of the possible cryptic species

The larval habitat of *An*. aff. *takasagoensis *was similar to that of *An. dirus *in southern Vietnam as described in Methods. However, the population density of *An*. aff. *takasagoensis *was extremely low so that we obtained only 11 larvae during the field collection for one week with seven staff members, even though we targeted on only this species. Moreover, the existence of the samples were localized; we found the samples from only one commune among four communes investigated.

In allozyme analyses of lactate dehydrogenase (LDH), glutamate-oxaloacetate transaminase (GOT), glucose phosphomutase (GPM), and glucose-6-phosphate dehydrogenase (G6PDH), other specimens of putative *An*. aff. *takasagoensis *also exhibited a different banding pattern from that of *An. dirus *in southern Vietnam (NTHN *et al*., unpublished data). In 2006, a 10 staff-member team of NIMPE were able to collect only five female adults in a buffalo hat over a one-month field-collection period in the same study area in Bắc Kạn (Le Xuan Hoi *et al*., personal communication). In 2007, Manh *et al*. collected two female adults of the putative cryptic species at the same buffalo hat. We succeeded to obtain partial *COI *(but not *ND6*) sequences of these two samples, and the haplotypes clustered with those of other *An*. aff. *takasagoensis *specimens analyzed in the present study (data not shown). These female adults seem to have been attracted by the buffalo. NDM, one of the co-authors of this study, failed in his attempt to feed an adult female with his blood in order to obtain progeny. This implies that the hypothetical cryptic species tends to be zoophilic, although in general, *An. dirus *is a highly anthropophilic species. This information reinforces our hypothesis that the mosquito population from northern Vietnam belongs to a cryptic species. Further investigations of such as karyotypes and polytene chromosome banding patterns are necessary to confirm whether *An*. aff. *takasagoensis *is a valid species.

## Conclusions

Morphological examination and molecular phylogenetic analyses of the members of the Leucosphyrus group in Vietnam suggested the existence of a cryptic species that is morphologically similar to, but genetically distant from both *An. dirus *and *An. takasagoensis*. We tentatively designated the species as *Anopheles *aff. *takasagoensis*. However, it was difficult to identify the species solely on the basis of morphological characteristics. Further studies on such as polytene chromosome banding patterns and karyotypes are necessary to confirm whether *An*. aff. *takasagoensis *is a valid species. Further studies on the (1) geographic distribution, which is potentially spreading along the Vietnam, China, Laos, and Myanmar borders; (2) morphological and ecological characteristics; and (3) vectorial capacity of this newly identified possible cryptic species of *An. dirus*, which is one of the most important malaria vectors in mainland Southeast Asia, are necessary for efficient malaria vector control in this region.

## Competing interests

The authors declare that they have no competing interests.

## Authors' contributions

KTT planned the study, conducted the field sampling of *An*. aff. *takasagoensis *in 2008 and molecular analyses, and drafted the manuscript. NTHN planned the study, conducted molecular analyses, and critically reviewed the manuscript. NTHB planned the study and critically reviewed the manuscript. TS directed the field sampling of *An*. aff. *takasagoensis *in 2008 through his expertise in the collection of larvae of the Dirus complex. He also conducted a preliminary investigation of the sampling field using GIS and critically reviewed the manuscript. MY contributed his expertise in molecular analyses and critically reviewed the manuscript. NDM planned the study; contributed his expertise in malaria vector control in Vietnam; collected, identified, and selected the samples; and critically reviewed the manuscript. MT planned the study, contributed his expertise in malaria vector control in Southeast Asia, and critically reviewed the manuscript. All authors read and approved the final manuscript.

## Supplementary Material

Additional file 1 **Alignment of partial sequences (221 bp) of the mitochondrial *COI *gene used in this study**. Every sequence is presented with the haplotype number and specific name that represents the sequence (c.f. Tables 1, 2 and 3). The consensus sequence indicates the most common bases for each site. Disagreement from the consensus sequence at each site is highlighted. Missing data are represented by an "N." Sequences of *Anopheles *aff. *takasagoensis *are surrounded by a frame. Click here for file

Additional file 2 **Alignment of partial sequences (349 bp) of the mitochondrial DNA *ND6 *gene used in this study**. Every sequence is presented with the haplotype number and specific name that represents the sequence (c.f. Tables 1, 2 and 3). The consensus sequence indicates the most common bases for each site. Disagreement from the consensus sequence at each site is highlighted. Missing data are represented by an "N." Sequences of *Anopheles *aff. *takasagoensis *are surrounded by a frame. Click here for file

Additional file 3 Alignment of concatenated partial sequences (570 bp in total) of the mitochondrial DNA *COI *and *ND6 *genes in FASTA format. Click here for file
