# Supplementary material for: Partial mitochondrial DNA sequences suggest the existence of a cryptic species within the Leucosphyrus group of the genus Anopheles (Diptera: Culicidae), forest malaria vectors, in northern Vietnam
Source: Parasit Vectors. 2010 Apr 30;3:41. doi: 10.1186/1756-3305-3-41 (PMC2881913; doi:10.1186/1756-3305-3-41)
Supplement: Additional file 2 — Alignment of partial sequences (349 bp) of the mitochondrial DNA ND6 gene used in this study. Every sequence is presented with the haplotype number and specific name that represents the sequence (c.f. Tables 1, 2 and 3). The consensus sequence indicates the most common bases for each site. Disagreement from the consensus sequence at each site is highlighted. Missing data are represented by an "N." Sequences of Anopheles aff. takasagoensis are surrounded by a frame. [file 1756-3305-3-41-S2.PDF]

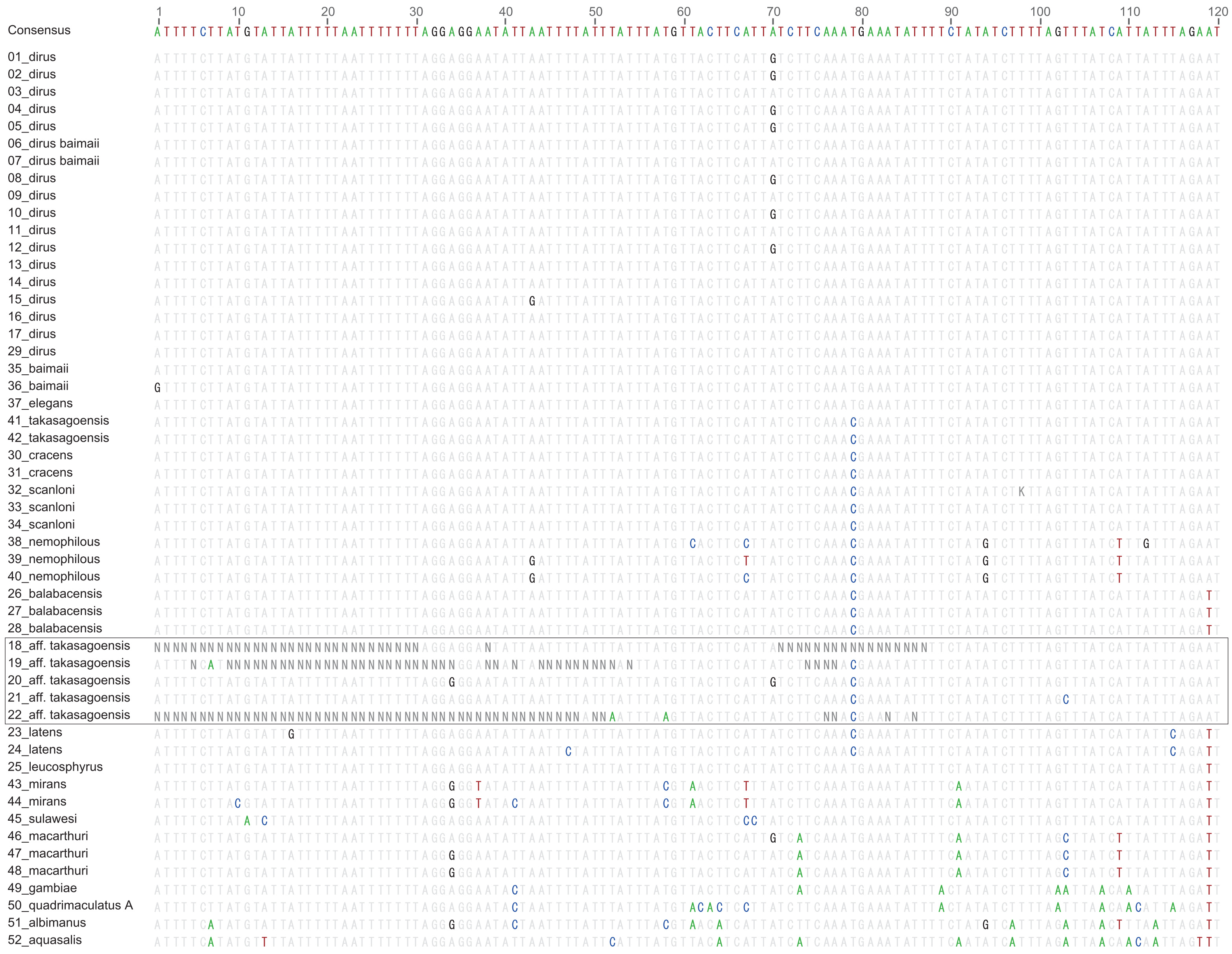
1 10 20 30 40 50 60 70 80 90 100 110 120
Consensus ATTTTCTTATGTATTATTTTTAATTTTTTTAGGAGGAATATTAATTTTATTTATTTATGTTACTTCATTATCTTCAAATGAAATATTTTCTATATCTTTTAGTTTATCATTATTTAGAAT
01_dirus
02_dirus
03_dirus
04_dirus
05_dirus
06_dirus baimaii
07_dirus baimaii
08_dirus
09_dirus
10_dirus
11_dirus
12_dirus
13_dirus
14_dirus
15_dirus
16_dirus
17_dirus
29_dirus
35_baimaii
36_baimaii
37_elegans
41_takasagoensis
42_takasagoensis
30_cracens
31_cracens
32_scanloni
33_scanloni
34_scanloni
38_nemophilous
39_nemophilous
40_nemophilous
26_balabacensis
27_balabacensis
28_balabacensis
18_aff. takasagoensis
19_aff. takasagoensis
20_aff. takasagoensis
21_aff. takasagoensis
22_aff. takasagoensis
23_latens
24_latens
25_leucosphyrus
43_mirans
44_mirans
45_sulawesi
46_macarthuri
47_macarthuri
48_macarthuri
49_gambiae
50_quadrimaculatus A
51_albimanus
52_aquasalis

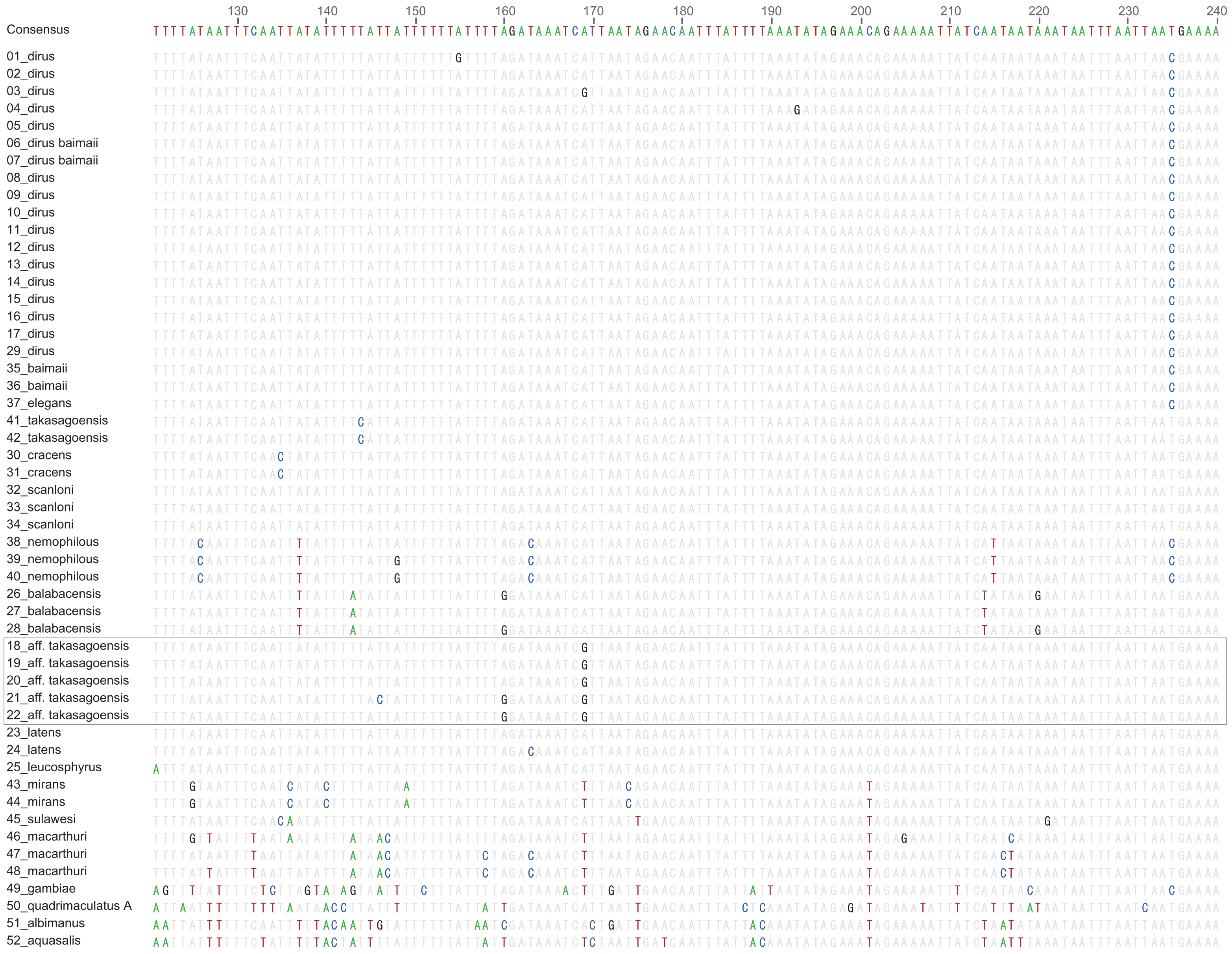

130
140
150
160
170
180
190
200
210
220
230
240
Consensus
01_dirus
02_dirus
03_dirus
04_dirus
05_dirus
06_dirus baimaii
07_dirus baimaii
08_dirus
09_dirus
10_dirus
11_dirus
12_dirus
13_dirus
14_dirus
15_dirus
16_dirus
17_dirus
29_dirus
35_baimaii
36_baimaii
37_elegans
41_takasagoensis
42_takasagoensis
30_cracens
31_cracens
32_scanloni
33_scanloni
34_scanloni
38_nemophilous
39_nemophilous
40_nemophilous
26_balabacensis
27_balabacensis
28_balabacensis
18_aff. takasagoensis
19_aff. takasagoensis
20_aff. takasagoensis
21_aff. takasagoensis
22_aff. takasagoensis
23_latens
24_latens
25_leucosphyrus
43_mirans
44_mirans
45_sulawesi
46_macarthuri
47_macarthuri
48_macarthuri
49_gambiae
50_quadrimaculatus A
51_albimanus
52_aquasalis

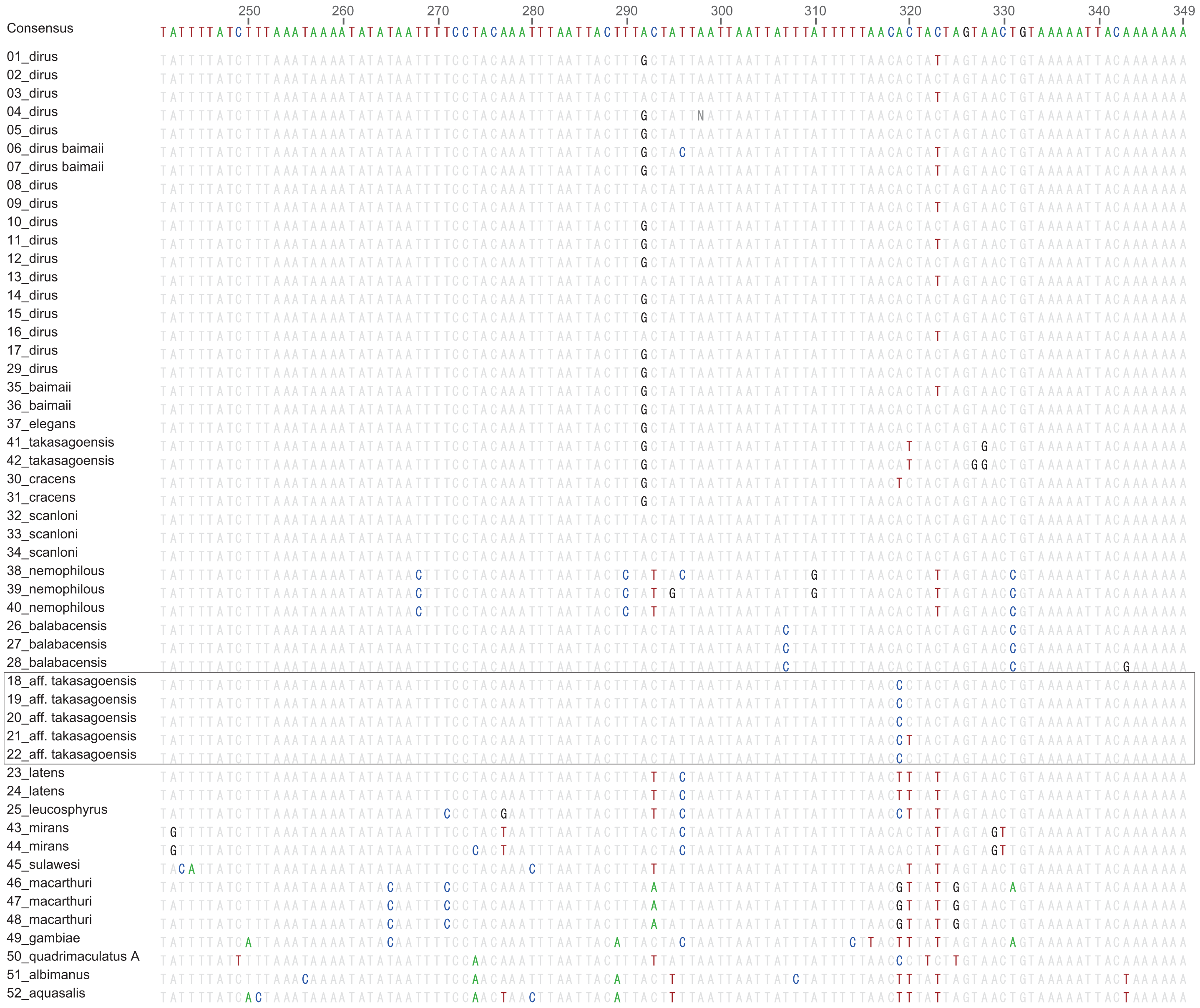
250 260 270 280 290 300 310 320 330 340 349
Consensus
TATTTTATCTTTAAATAAAATATATAATTTTCCTACAAATTTAATTACTTTACTATTAATTAATTATTTATTTTTAACACTACTAGTAACTGTAAAAATTACAAAAAAA
01_dirus
02_dirus
03_dirus
04_dirus
05_dirus
06_dirus baimaii
07_dirus baimaii
08_dirus
09_dirus
10_dirus
11_dirus
12_dirus
13_dirus
14_dirus
15_dirus
16_dirus
17_dirus
29_dirus
35_baimaii
36_baimaii
37_elegans
41_takasagoensis
42_takasagoensis
30_cracens
31_cracens
32_scanloni
33_scanloni
34_scanloni
38_nemophilous
39_nemophilous
40_nemophilous
26_balabacensis
27_balabacensis
28_balabacensis
18_aff. takasagoensis
19_aff. takasagoensis
20_aff. takasagoensis
21_aff. takasagoensis
22_aff. takasagoensis
23_latens
24_latens
25_leucosphyrus
43_mirans
44_mirans
45_sulawesi
46_macarthuri
47_macarthuri
48_macarthuri
49_gambiae
50_quadrimaculatus A
51_albimanus
52_aquasalis
